# The Impact of Pigment-Epithelium-Derived Factor on MCF-7 Cell Metabolism in the Context of Glycaemic Condition

**DOI:** 10.3390/pharmaceutics15082140

**Published:** 2023-08-14

**Authors:** Raziyeh Abooshahab, Kourosh Hooshmand, Hani-Al Salami, Crispin R. Dass

**Affiliations:** 1 Curtin Medical School, Curtin University, Bentley, WA 6102, Australia; raziyeh.abooshahab@postgrad.curtin.edu.au (R.A.); hani.al-salami@curtin.edu.au (H.-A.S.); 2 Curtin Health Innovation Research Institute, Curtin Medical School, Curtin University, Bentley, WA 6102, Australia; 3 System Medicine, Steno Diabetes Center Copenhagen, 2730 Copenhagen, Denmark; kourosh.hooshmand@gmail.com; 4 Biotechnology and Drug Development Research Laboratory, Curtin Health Innovation Research Institute, Bentley, WA 6102, Australia

**Keywords:** breast cancer, cancer metabolism, metabolomics, glycaemia, PEDF

## Abstract

Studies have demonstrated that pigment-epithelium-derived factor (PEDF) is a robust inhibitor of tumour growth and development, implying that this may serve as a promising target for therapeutic intervention. However, the precise impact of PEDF on cancerous cell metabolic pathways remains uncertain despite ongoing research. In this light, this study aimed to employ a metabolomics approach for understanding the metabolic reprogramming events in breast cancer across different glycaemic loads and their response to PEDF. Gas chromatography-quadrupole mass spectrometry (GC/Q-MS) analysis revealed metabolic alterations in ER^+^ human cell line MCF-7 cells treated with PEDF under varying glycaemic conditions. The identification of significantly altered metabolites was accomplished through MetaboAnalyst (v.5.0) and R packages, which enabled both multivariate and univariate analyses. Out of the 48 metabolites identified, 14 were chosen based on their significant alterations in MCF-7 cells under different glycaemic conditions and PEDF treatment (*p* < 0.05, VIP > 0.8). Dysregulation in pathways associated with amino acid metabolism, intermediates of the TCA cycle, nucleotide metabolism, and lipid metabolism were detected, and they exhibited different responses to PEDF. Our results suggest that PEDF has a diverse influence on the metabolism of MCF-7 cells in both normo- and hyperglycaemic environments, thereby warranting studies using patient samples to correlate our findings with clinical response in the future.

## 1. Introduction

Breast cancer (BC) accounts for nearly 25% of all cancers diagnosed in women [1]. Based on molecular characteristics, BC can be divided into several subtypes, each with distinct biological features and clinical outcomes. The most widely used classification system is the one developed by the Cancer Genome Atlas (TCGA) network [2], which includes the following subtypes: luminal-like tumours, which consist of luminal-A, estrogen receptor ^+^/progesterone receptor ^+^/human epidermal growth factor receptor 2^−^/(ER^+^/PR^+^/HER2^−^) and luminal-B (ER^+^/PR^−^/HER2^+^) tumours, HER2-enriched breast cancers, which exhibit overexpression of the receptor tyrosine-protein kinase Erb-B2 oncogene, and basal-like tumours, that are heterogeneous and a more aggressive subtype [2,3]. Despite notable advancement in the management of BC in recent decades, mainly due to better earlier detection techniques, the efficacy of therapeutic interventions remains suboptimal and is contingent upon various factors. The limited success in managing BC is, to a certain extent, attributable to a lack of pathophysiological understanding of the disease, which translates to subpar individualised treatment and unfavourable clinical outcomes [4]. Consequently, developing novel therapeutic modalities for BC is critical to improving BC patient outcomes.

Cancerous cells exhibit a unique metabolic phenotype that confers the ability to sustain high energetic demands of vigorous cellular proliferation and growth [5]. A notable characteristic of cancerous cells is their augmented reliance on glucose as an energy substrate, a phenomenon commonly referred to as the Warburg effect, characterised by elevated glucose uptake and glycolytic activity, even under aerobic conditions [6]. This starkly contrasts the oxidative-phosphorylation-dependent energy production mechanism utilised by normal cells. Furthermore, cancerous cells exhibit an altered metabolic profile characterised by the activation of biosynthetic pathways that facilitate the generation of fundamental macromolecules, such as nucleotides and lipids, crucial for supporting the cellular proliferation and progression requisite for tumourigenesis [7,8]. A growing body of evidence suggests a link between hyperglycaemia (elevated blood sugar levels) and BC, in which tumours may be more likely to grow and spread in a high-glucose environment [9,10]. Several studies have shown that hyperglycaemia can induce inflammation, oxidative stress, and hormonal imbalances, contributing to the development and progression of BC [10,11]. Additionally, insulin resistance, a metabolic dysfunction often concomitant with hyperglycaemia, has been linked to an increased risk of BC [10]. By unravelling the underlying biology of BC in this area, practitioners can then develop therapeutic strategies tailored to the specific characteristics of each patient’s tumour.

Pigment-epithelium-derived factor (PEDF) is a secreted glycoprotein of the serpin (serine protease inhibitor) superfamily, which was initially identified as a potent angiogenic inhibitor secreted by retinal pigment epithelium (RPE) cells [12]. PEDF exhibits a plethora of molecular activities, including neurotrophic, anti-inflammatory, anti-oxidative, and anti-tumour functions [13,14]. These functions are mediated through its interaction with various receptors, such as laminin receptor (LR) [15] and PEDF receptor (PEDF-R) [16]. Notably, PEDF has been proposed to modulate the lipolytic pathway through its interaction with its receptor, adipose triglyceride lipase (ATGL), suggesting its involvement in regulating lipid metabolism and energy balance [17]. Furthermore, PEDF’s influence on this pathway has been associated with improved insulin resistance and enhanced metabolic efficiency [17,18]. Numerous studies have suggested that a reduction in PEDF expression is linked to increased poor prognosis and tumour aggressiveness in various types of cancer [13,19] and have consistently demonstrated the downregulation of PEDF in BC cells [13]. Thus, the multifaceted role of PEDF in cancer has generated considerable interest among researchers, highlighting the potential for PEDF-based therapeutics in cancer treatment in the future. Although previous research has acknowledged PEDF’s multifunctional properties and its potential role as a metabolic regulator, the specific interplay between PEDF, glycaemic conditions, and tumour cell metabolism remains limited in terms of mechanistic elucidation.

Metabolomics, a rapidly evolving field of omics sciences, plays a pivotal role in cancer research by providing valuable insights into the metabolic alterations associated with tumourigenesis and disease progression [20]. Untargeted metabolomics is an unbiased approach that allows for discovering novel metabolic alterations [21]. It does not rely on predefined targets, enabling the detection of unexpected or previously unknown metabolic changes [20,21] and identifying new therapeutic targets or pathways. In a recently published study, we utilised gas chromatography-mass spectrometry (GC/MS) to point out metabolic alterations in the triple-negative breast cancer (TNBC) cell line (MDA-MB-231) after being subjected to glycaemic loading in response to PEDF [22]. However, to gain a comprehensive understanding of the metabolic reprogramming occurring in each BC subtype under glycaemic loading and to assess the effectiveness of PEDF, we performed our experiment on the MCF-7 cell line to the intricate molecular pathways and metabolic adaptations by employing untargeted metabolomics. The dissimilarities in both the phenotypic and genotypic characteristics of the MCF-7 and MDA-MB-231 cell lines make them ideal subjects for individualised experimentation to explore the complex interplay of the metabolic pathways involved in tumourigenesis and progression as well as their distinct metabolic responses to PEDF. Unravelling the mechanisms governing the reprogramming of metabolic pathways associated with PEDF treatment in BC cells in the context of glycaemic conditions can open up new avenues for further targeted research and ultimately enhance the development of more effective cancer treatments.

## 2. Materials and Methods

### 2.1. Reagents and Materials

The recombinant PEDF was supplied by MD Bioproducts (Bethesda, MD, USA). The MCF-7 was from the American Tissue Culture Collection, ATCC (Manassas, VA, USA). Dulbecco’s modified Eagle medium (DMEM), foetal bovine serum (FBS), and antibiotic/antimycotic were all purchased from Sigma-Aldrich, Saint Louis, MO, USA. HPLC-grade isopropanol (IPA), methanol (MeOH), and water (H_2_O) were obtained from Sigma-Aldrich, along with alkanes, 4,4′-dibromooctafluorobiphenyl, and hexane. Derivatisation reagents including methoxyamine (MOX), trifluoroacetamide (MSTFA), and 1% trimethylsilyl chloride (TMCS) were purchased from Thermo Fisher Scientific, Waltham, MA, USA.

### 2.2. Cell Cultivation

The human luminal-A (ER^+^/PR^+/−^/HER2^−^) breast adenocarcinoma cell line (MCF-7) was cultured in DMEM with 10% foetal bovine serum (FBS) and 1% antibiotics and antimycotics under normal glucose conditions (5 mM). The cultures were grown to 80% confluence under controlled conditions at 37 °C with 5% CO_2_ and passaged weekly following the established ATCC protocols. For the metabolomics analysis, the MCF-7 cells were seeded in 24-well plates at a density of 4 × 10^5^ cells/well in two distinct media groups with varying glucose concentrations (5 mM and 25 mM). After the cells were cultured for 24 h, they were exposed to a physiologically normal concentration of PEDF (100 nM) [23] and then incubated overnight. Subsequently, the cells underwent trypsinisation and were centrifuged at 700× *g* for 5 min. The culture medium was discarded, and the obtained pellets were promptly frozen at −80 °C until sample preparation. The experiment was carried out with six replicates per media condition.

### 2.3. Sample Preparation for Metabolomics Analysis

Metabolite extraction and derivatisation were performed following a previously described method [22]. In brief, 500 µL of cooled protein precipitation solvent containing methanol/isopropanol/water was added to each sample tube. After mixing and chilling for 20 min at 4 °C, the resulting mixtures were centrifuged at 21,952× *g* for 15 min at 4 °C. Finally, the supernatants containing the cell metabolites were subjected to evaporation to dryness under a nitrogen stream. The dried residues were derivatised using two steps as follows: methoxyamination was performed by adding 30 μL of methoxyamine hydrochloride and heating for an hour at 60 °C. This preceded the addition of MSTFA as trimethylsilylation reagent containing 1% trimethylchlorosilane (TMCS) and a standard mixture of the alkane retention index (50 μL). The mixture was then incubated for 20 min at 45 °C to allow the reaction to take place. Following this, each sample was dissolved with 20 µL of an injection standard that contained 4,4′-dibromooctafluorobiphenyl at a concentration of 10 mg/L in hexane. The supernatant of each sample was immediately transferred to autosampler GC/MS glass vials for GC/MS analysis.

### 2.4. Metabolome Profiling Using GC/Q-MS

An Agilent 5977B MSD/Agilent 8860 GC system fitted with a Restek Rxi-5-ms column (30 m length × 0.25 mm internal diameters (id); 0.25 μm film) was used to analyse the derivatised samples. Approximately 1 µL of each sample was injected at a split ratio of 1:1 in random order. The chromatographic method was conducted at a constant flow rate of helium 1 mL/min as the carrier gas, ramping from 20 °C/min to 320 °C, and then holding at 320 °C for 5 min. The MS source, transfer line, and quadrupole temperature were set at 150 °C, 290 °C, and 250 °C, respectively, operating in electron ionisation mode at −70 eV. After a 5.4 min solvent delay, mass spectrometry data were collected at a scan rate of 20 spectra/sec within the range of *m*/*z* 50–600.

### 2.5. Data Processing and Statistical Analysis

The data were processed using MS-DIAL (version 4.9) to create a data matrix consisting of InChIKey, peak intensity, and the original dataset’s average retention time (RT). Metabolite names were assigned to the GC/MS spectra based on two parameters: mass spectral similarity and retention indices (calculated using a mixture of alkanes). To confirm the identified metabolites, the GC/MS spectra were compared to several mass spectral libraries, including Fiehn library, MassBank, Golm DB, GNPS, and HMDB. The peak intensity was normalised by sum and scaled using autoscaling with MetaboAnalyst (v5.0) to achieve normal distribution. Multivariate and univariate statistics and visualisation were performed with supervised partial least squares discriminant analysis (PLS-DA) and one-way analysis of variance (ANOVA). The data were displayed as a boxplot by applying the R packages “ggpubr” and “tidyverse”. An investigation into the primary biological pathways was conducted via an enrichment pathway analysis using MetaboAnalyst (v5.0), with a significance threshold of *p*-value < 0.05 and FDR < 0.1.

## 3. Results

### 3.1. Metabolic Profile among the Groups

**Figure 1:**
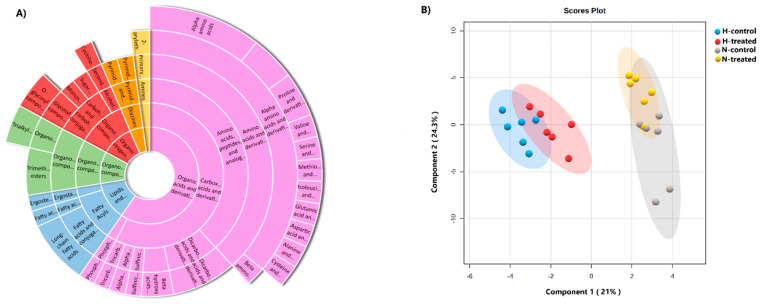
(**A**) The sunburst plot displaying the entire range of metabolomes identified in this study, including amino acids, lipids, and other classes, along with their corresponding metabolites and conjugates. (**B**) PLS-DA plot of MCF-7 cells under glycaemic loading with and without PEDF created with 95% confidence and cross-validated R2Y = 0.98122 and Q2 = 0.80111 coefficients.

The data were processed by MS-DIAL, resulting in 356 compounds, out of which 48 metabolites were considered reliable for further analysis (Table S1), and then categorised into different levels formed from the ClassyFire system [24]. Metabolites at the subclass level were abundant, with amino acids, peptides, and analogues accounting for a significant portion, along with dicarboxylic acids and derivatives (Figure 1A). The complete names and classes of the metabolites are set out in Table S2.

**Figure 2:**
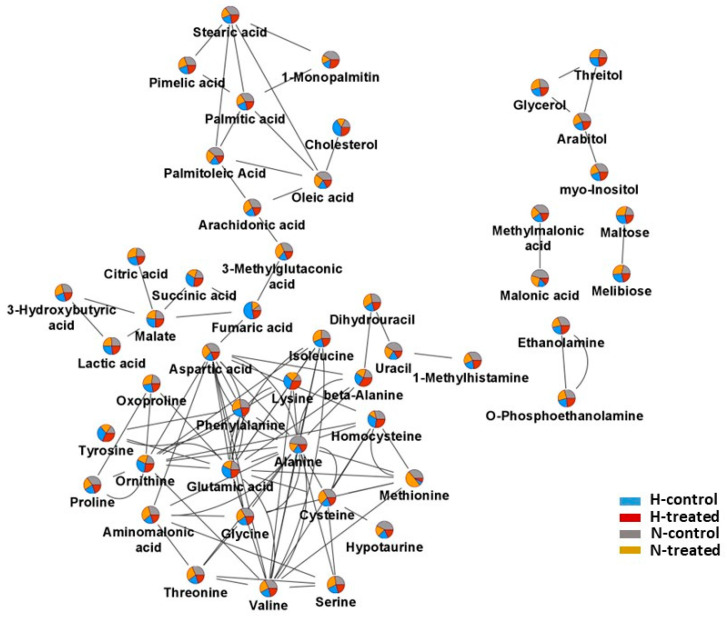
Molecular network of all detected metabolites using GC/MS. Pie charts displaying the distribution of metabolite intensities under the high-glucose (H-control: blue; H-treated: red) and normal-glucose (N-control: grey; N-treated: dark yellow) conditions.

A supervised PLS-DA model score plot showed a perfect separation among the groups (Figure 1B). The cross-validation and the permutation revealed that the model was significant (Figure S1A,B). To conduct a comprehensive analysis of the metabolome, molecular networks were employed. Figure 2 displays nodes represented as pie charts depicting the metabolic changes in MCF-7 cells in response to glycaemic loading and PEDF treatment.

### 3.2. Response of MCF-7 Metabolomes to PEDF under Glycaemic Loading

**Figure 3:**
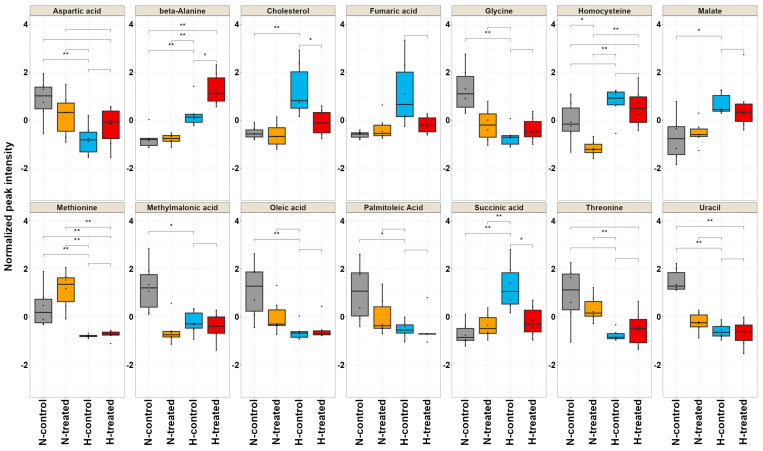
The boxplots display the distribution of 14 metabolites that exhibited the highest significance (*p*-values < 0.05 and VIP scores > 0.8) in the analysis of variance. These boxplots allow for comparing the four groups: normal and high-glucose conditions with and without PEDF. On the *x*-axis, each group is represented by individual metabolites, while the *y*-axis indicates the normalised peak intensity. Metabolites showing significant differences were calculated using Tukey’s Honestly Significant Difference (TukeyHSD) test and indicated as (*) *p* ≤ 0.05, and (**) *p* ≤ 0.01. The key for the groups is as follows: H represents high-glucose (H-control: blue; H-treated: red) and N represents normal-glucose (N-control: grey; N-treated: dark yellow).

Using one-way ANOVA analysis (*p*-values < 0.05, FDR q < 0.05) coupled with a multivariate test with a VIP score of ≥0.8 revealed that out of 48 metabolites, 14 metabolites exhibited substantial differences among the groups (Table 1). To visualise the differences amongst the four groups (normal and high-glucose conditions with and without PEDF), boxplots were constructed for the most significant metabolites using data from the ANOVA analysis (Figure 3). These findings indicated that certain metabolites involved in lipid metabolism, such as cholesterol, oleic acid, and palmitoleic acid, were reduced under normal and high-glucose conditions after exposure to PEDF. Cholesterol exhibited the most pronounced changes. The metabolite levels that pertain to amino acid metabolism, including glycine, methionine, aspartic acid, beta-alanine, and threonine, showed considerable alterations among the groups. Methionine presented notable changes, decreasing substantially under high-glucose conditions and increasing under both normal and high-glucose conditions after exposure to PEDF. Tricarboxylic acid cycle (TCA) cycle intermediates, including malate, succinic acid, and fumaric, displayed distinct patterns in their intensities after exposing the cells to PEDF under glycaemic loading. PEDF’s impact on homocysteine and methylmalonic acid levels was statistically significant, influencing their response by the glucose level.

### 3.3. Pathway Analysis

**Figure 4:**
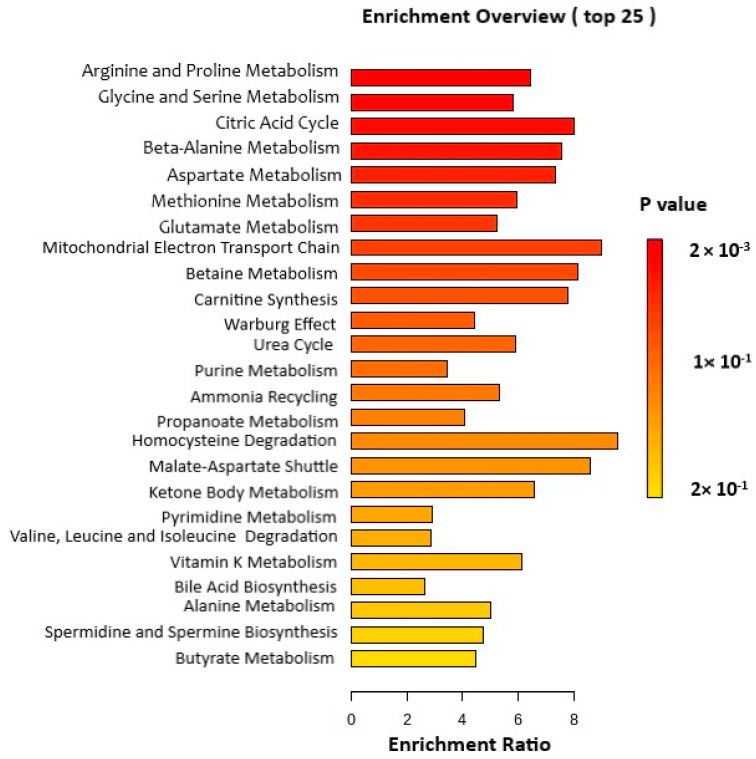
A metabolite set enrichment analysis (MSEA) based on differentially altered metabolites identified among the groups. MSEA was used to detect significantly enriched metabolic pathways among the groups.

MetaboAnalyst (v.5.0) was used to perform an enrichment analysis of the 14 critical metabolites. The findings indicated that PEDF had a notable impact on three major metabolic pathways. The first pathway affected by PEDF was arginine and proline metabolism, as evidenced by changes in glycine, fumaric acid, aspartic acid, and succinic acid. The second pathway influenced by PEDF was glycine and serine metabolism, which exhibited alterations in glycine, threonine, methionine, and homocysteine. The third significant pathway affected by PEDF was the citric acid cycle with amended metabolites such as fumaric acid, malate, and succinic acid (Figure 4). All of the enriched pathways can be found in Table S3.

## 4. Discussion

Hyperglycaemia, a metabolic diabetes, has been implicated in recent decades as a risk factor for breast cancer [9], which can not only increase the mortality and incidence of BC but also highlights the negative impact on the effectiveness of chemotherapy and the potential development of chemoresistance [10]. Hence, gaining a comprehensive understanding of the various types of metabolic reprogramming in the field is crucial to create targeted treatments that tackle the altered pathways in BC.

It has been demonstrated that PEDF is associated with metabolic disorders [18]. On the other hand, various studies have reported reduced PEDF expression in BC cells [13]. Nevertheless, no conclusive evidence supports the notion that PEDF plays a specific role in the metabolic alteration of BC cells under glycaemic status. Our group recently conducted a study utilising GC/MS analyses to investigate metabolic shifts in the TNBC cell line (MDA-MB231) in response to glycaemic loading and PEDF exposure [22]. The results showed that the Warburg effect in MDA-MB-231 was affected by PEDF regardless of glucose level. To gain a more comprehensive understanding of the metabolic reprogramming in each subtype of BC with distinct metabolism and to evaluate the efficacy of PEDF, we conducted additional experiments on the MCF-7 cell line to examine changes in metabolism in response to glycaemic loading and PEDF exposure.

The metabolomic analysis conducted in this study revealed significant alterations in the metabolic profile of MCF-7 breast cancer cells following PEDF treatment under normal and high-glucose conditions. Several metabolites were found to be differentially regulated by PEDF treatment, including uracil, beta-alanine, methionine, glycine, succinic acid, cholesterol, homocysteine, methylmalonic acid, oleic acid, fumaric acid, threonine, malate, palmitoleic acid, and aspartic acid. These metabolites play crucial roles in various metabolic pathways, including nucleotide, amino acid, lipid, and energy metabolism.

The biosynthesis of nucleotides plays a vital role in the metabolic processes necessary for tumour cell replication [25]. Accordingly, the replication of malignant cells hinges on their ability to synthesise DNA and RNA through nucleotide metabolism, thereby enabling them to undergo self-proliferation without any regulatory constraints [25,26]. In the present study, the observed decrease in uracil levels in the high-glucose groups suggests that elevated glucose levels may negatively impact nucleotide metabolism. This finding is remarkable as it indicates a potential metabolic vulnerability in tumour cells exposed to high-glucose environments. Additionally, the study showed that exposure to PEDF further reduced uracil levels under both normal and high-glucose conditions. Notably, the more pronounced decrease in uracil levels under normal glucose conditions suggests that PEDF may have a greater impact on nucleotide metabolism in a non-diabetic or normoglycemic environment. Under high-glucose conditions, different metabolic pathways may play more prominent roles, potentially compensating for the effects of PEDF on uracil metabolism and resulting in a more modest reduction in uracil levels. This also accords with our earlier observations, which showed changes of uracil in MDA-MB231 cells under both normo- and hyperglycemic conditions; however, the alteration of it was not significant [22]. This finding is particularly interesting because it highlights the capability of PEDF as a nucleotide metabolism modulator and suggests its potential therapeutic relevance in targeting tumour cell replication. The metabolism of uracil in humans is a multifaceted and highly regulated process that involves numerous enzymatic reactions, including uridine phosphorylase 2 [27], uridine kinase [28], and uracil phosphoribosyltransferase [29], which ultimately convert uracil into uridine monophosphate (UMP), a precursor for RNA synthesis. In cancer cells, the metabolism of uracil is often altered due to changes in gene expression or mutations in key enzymes, resulting in the accumulation of uracil and its derivatives [28,30]. Considering the role of nucleotide metabolism in cancer cell proliferation and the observed effects of glucose and PEDF on uracil levels, these findings provide insights into the metabolic regulation of tumour cells and raises intriguing questions about the underlying mechanisms involved.

Amino acid metabolism is one of the key pathways that is altered in cancer cells, and changes in the levels of specific amino acids have been linked to tumour cell growth and survival [31]. The present study showed that the metabolism of approximately six amino acids changed among groups with different responses to PEDF under glycaemic conditions. Methionine, glycine, and beta-alanine showed the most significant differences. Glycine is a non-essential amino acid that plays an essential role in various cellular processes, including protein synthesis, energy metabolism, and redox regulation [32]. In our study, in the high-glucose group, a decreased level of glycine compared to the normal glucose group was noted. These changes regarding glycine levels corroborate the findings of our previous study performed on MDA-MB-231 [22]. The decrease in glycine levels under high-glucose conditions may be due to a combination of reactive oxygen species (ROS)-mediated oxidation, glycation end products (AGEs) formation [33], and increased demand due to mammalian target of rapamycin (mTOR) activation [34]. In addition, after exposing cells to PEDF, glycine levels decreased and slightly increased under normal and high-glucose conditions, respectively. This suggests that PEDF may have a protective effect on glycine depletion induced by high glucose levels, potentially through inhibition of the mTOR pathway or by mitigating oxidative stress.

The reduction in methionine levels observed in MCF-7 cells under high-glucose conditions may be attributed to the upregulation of the transsulfuration pathway, which diverts the methionine pool towards cysteine synthesis [35,36]. This diversion is mediated by cystathionine-β-synthase (CBS) activation due to increased cellular energy levels [36]. Moreover, we observed elevated levels of homocysteine under normal conditions; a sulphur-containing amino acid formed during the metabolism of methionine to cysteine [37,38]. Consequently, a decrease in methionine and an increase in homocysteine indicate dysregulation in methionine metabolism in response to the glucose-rich environment in ER^+^ human BC. Elucidating the precise mechanisms underlying these changes is challenging due to metabolism’s complexity and other factors’ influence. There are similarities between the alteration of methionine by PEDF in the glycaemic environment in this study and the one that our group performed on MDA-MB-231; however, the changes were not significant [22]. Exposure to PEDF seemed to regulate methionine and homocysteine levels under both normal and high-glucose conditions. The slight increase in methionine levels under high-glucose conditions after PEDF exposure could be attributed to the potential activation of enzymes involved in methionine synthesis, partially compensating for the earlier observed decrease in methionine levels. Further investigation allows us to gain a deeper understanding of how PEDF may impact this particular pathway.

Our experiment uncovered a noteworthy observation pertaining to the beta-alanine levels. Beta-alanine is a non-proteinogenic amino acid produced in the human body [39] from the breakdown of pyrimidines, the decarboxylation of l-aspartate by intestinal microbes, and transamination [40,41]. Beta-alanine plays an essential role in the synthesis of carnosine, a dipeptide found primarily in muscle tissue [42]. Carnosine contributes to various physiological processes in the body, including buffering acidity in muscle cells, reducing oxidative stress, and regulating glucose metabolism [43]. Additionally, carnosine has been found to inhibit the proliferation of breast, ovarian, colon, and leukemic cancer cells [44]. Interestingly, exposure to PEDF increased beta-alanine levels under both normal and high-glucose conditions in ER^+^ human BC. This implies that PEDF might directly target beta-alanine biosynthetic pathways or interact with upstream regulators, circumventing the influence of glucose availability. This finding can potentially be elucidated by the biochemical conversion of uracil into beta-alanine, whereby the administration of PEDF triggered a decline in uracil concentration followed by an elevation in beta-alanine levels, facilitating carnosine biosynthesis. The elevated beta-alanine levels induced by PEDF may have broader implications in the context of cancer biology and metabolic disorders. Beta-alanine, as a component of carnosine, can modulate intracellular pH and act as an antioxidant [43], potentially influencing cancer cell proliferation, survival, and response to oxidative stress [42]. Moreover, dysregulation of beta-alanine metabolism has been linked to metabolic disorders, suggesting that the PEDF-mediated increase in beta-alanine levels may have implications for metabolic homeostasis. As we delve deeper into our investigations, our understanding of how PEDF can regulate metabolic processes through its impact on beta-alanine continues to expand.

Another important finding was the significant changes in the metabolites associated with the TCA cycle, particularly malate, fumaric acid, and succinic acid. All three increased under high-glucose conditions, and after exposure to PEDF, their levels increased under normal conditions and decremented under high-glucose conditions. In concordance with the present results, our previous study demonstrated the same trends regarding the mentioned metabolites in MDA-MB-231 in that the malate levels showed more significant differences between them [22]. Under high-glucose conditions, the increased availability of pyruvate and other TCA cycle intermediates may lead to an increased cycle rate, resulting in the production of excessive fumaric acid, succinic acid, and malate [45,46]. Fumaric acid can stimulate the Keap1/Nrf2 pathway, leading to the upregulation of antioxidant genes and cytoprotective mechanisms, thereby promoting tumour cell survival [47]. Known to be a prolyl hydroxylases inhibitor, succinic acid is able to stabilise hypoxia-inducible factors (HIFs) and activate genes involved in angiogenesis and energy metabolism [48]. Furthermore, malate generation in cancer cells serves as an effective mechanism for supporting glycolysis by promoting NADPH synthesis, an important cofactor for antioxidant defences and other cellular processes [49,50]. These changes in fumaric acid, succinic acid, and malate levels in response to PEDF and high-glucose conditions reflect the complex interplay between different metabolic pathways and cellular responses to these conditions. Nevertheless, these findings suggest that PEDF can protect the balance of TCA cycle metabolism regardless of glucose conditions.

The present study observed a noteworthy alteration in lipid metabolism, specifically concerning cholesterol, which agrees with our previous work on MDA-MB-231. Cholesterol plays an integral role in cell membranes [51] and the synthesis of certain hormones that regulate cell growth and functions [52]. Cancer cells heavily rely on cholesterol for their growth and proliferation [53]. PEDF can modulate different signaling pathways involved in lipid metabolism. The effect of PEDF on lipid metabolism and its precise mechanisms are still not fully understood. However, previous studies has shown that PEDF can enhance lipolysis by binding to the adipose triglyceride lipase (ATGL), a key enzyme responsible for the breakdown of triglycerides [54]. Our study demonstrates that high-glucose levels significantly increased cholesterol levels compared to normal conditions. Nevertheless, the administration of PEDF in both situations ultimately leads to decreased cholesterol levels. This compelling evidence suggests that PEDF might profoundly impact lipid metabolism during cellular stress.

However, despite their similarities in metabolite alterations under normal and high-glucose conditions and responses to PEDF, MCF-7 and MDA-MB-231 exhibit distinct differences in their metabolic profiles. The metabolic disparities between MCF-7 and MDA-MB-231 cells are likely attributed to their different genetic backgrounds and molecular characteristics [55]. MCF-7 cells are estrogen receptor positive and represent a luminal subtype of breast cancer, while MDA-MB-231 cells are triple negative and belong to the basal-like subtype. These divers’ molecular profiles could lead to disparities in the expression and activity of metabolic enzymes and pathways and subsequently influence their response to glucose availability and the modulation of metabolic pathways by PEDF. As an example, in our previous study, we observed a significant impact of PEDF on metabolites associated with the Warburg effect in MDA-MB-231 cells, specifically lactate and glucose-6-phosphate (G6P) [22]. In contrast, in the case of MCF-7 cells, G6P was not identified as a metabolite, suggesting a differential metabolic response between these cell lines. Interestingly, the effect of PEDF on lactate levels in MCF-7 cells mirrored that observed in MDA-MB-231 cells, although the changes were not statistically significant. On the other hand, our findings indicate that in MCF-7 cells, PEDF exerts a more pronounced effect on metabolic pathways associated with arginine, proline, glycine, serine, and the TCA cycle, as evidenced by alterations in metabolite levels. This starkly contrasts with the impact of PEDF on metabolic pathways in MDA-MB-231 cells, highlighting a differential metabolic response to PEDF between the two breast cancer cell lines.

## 5. Conclusions

After considering the original objective of this study, it is now possible to state that this finding yielded valuable insights into the link between metabolic processes and glycaemic conditions in BC, specifically the luminal-A (ER^+^/PR^+/−^/HER2^−^) subtype, as well as the significant impact of PEDF on metabolic pathways. The metabolomics data analysis demonstrates that PEDF has a diverse impact on the metabolism of MCF-7 cells under both normo- and hyperglycaemic conditions. More importantly, this study has identified new metabolic markers that are heavily influenced by PEDF, which can play a crucial role in regulating the metabolism of BC cells. Understanding how PEDF affects cellular metabolism provides insights into the complex regulatory mechanisms that cells employ to adapt to changing environmental conditions, such as glucose availability. This knowledge contributes to our understanding of cellular homeostasis and metabolic plasticity. PEDF has been shown to modify the levels of metabolites in essential metabolic pathways such as the TCA cycle, lipid metabolism, and amino acid metabolism. While there are notable similarities in the alteration of metabolites and PEDF responses between MCF-7 and MDA-MB-231 cells, their distinct metabolic profiles highlight the need for a comprehensive understanding of the metabolic heterogeneity within breast cancer subtypes under stress conditions. Unraveling the complexities of their divergent metabolism could provide valuable insights into their respective tumour biology and aid in developing personalised treatment approaches for breast cancer patients. The observed impact of PEDF on diverse metabolic pathways in BC cells raises intriguing questions about the underlying mechanisms. Further investigations into the specific molecular interactions and signaling cascades involved in PEDF-mediated metabolic regulation can provide valuable mechanistic insights. Understanding these mechanisms can uncover novel targets for therapeutic intervention and enhance our knowledge of fundamental cellular processes. Hence, more research must be conducted to fully discover primary mechanisms of these effects, especially at the protein level, to enhance the accuracy of initial therapeutic selection systems and identify new targets to improve breast cancer treatment.

## Figures and Tables

**Table 1:** Significant alterations in metabolites under normal and high-glucose conditions, both with and without PEDF (one-way ANOVA and Tukey’s HSD tests).

Metabolites	VIP	One-Way ANOVA		Multiple Comparisons Tukey HSD (^a^ *p*-Value)					
		^a^ *p*-Value	FDR	HT vs. HC	NC vs. HC	NT vs. HC	NC vs. HT	NT vs. HT	NC vs. NT
**Uracil**	1.0007	1.07 × 10^−7^	5.14 × 10^−6^	0.982	0.000	0.525	0.000	0.325	0.000
**beta-Alanine**	1.587	1.39 × 10^−6^	3.33 × 10^−5^	0.012	0.012	0.011	0.000	0.000	1.000
**Methionine**	2.1201	2.56 × 10^−5^	0.000409	0.999	0.011	0.000	0.015	0.000	0.188
**Glycine**	0.97332	0.000313	0.003589	0.851	0.000	0.573	0.002	0.959	0.007
**Succinic acid**	1.643	0.000448	0.003589	0.008	0.000	0.003	0.584	0.970	0.836
**Cholesterol**	1.8464	0.000449	0.003589	0.014	0.001	0.001	0.715	0.568	0.995
**Homocysteine**	1.9154	0.000654	0.004486	0.946	0.246	0.001	0.479	0.002	0.059
**Methylmalonic acid**	1.5986	0.002992	0.015957	0.966	0.010	0.833	0.003	0.981	0.001
**Oleic acid**	1.0777	0.006105	0.041381	0.994	0.004	0.490	0.007	0.644	0.089
**Fumaric acid**	1.4726	0.004588	0.022023	0.037	0.005	0.019	0.793	0.989	0.929
**Threonine**	1.4385	0.005339	0.023298	0.931	0.007	0.104	0.027	0.290	0.596
**Malate**	1.4995	0.010921	0.040484	0.999	0.036	0.084	0.049	0.112	0.974
**Aminomalonic acid**	1.7133	0.010964	0.040484	0.631	0.052	0.013	0.414	0.148	0.911
**Palmitoleic Acid**	0.96085	0.012301	0.042175	1.000	0.019	0.675	0.020	0.685	0.180
**Aspartic acid**	1.2957	0.014993	0.047976	0.673	0.011	0.168	0.116	0.737	0.548

Key: ANOVA, analysis of variance; VIP, variation important in the projection; FDR, false discovery rate; HT, high-glucose treated; HC, high-glucose control; NT, normal-glucose treated; NC, normal-glucose control. ^a^ *p*-value < 0.05 is considered statistically significant based on the one-way ANOVA and Tukey’s post hoc test.

## Data Availability

All data generated or analyzed during this study are included in this article and its Supplementary Materials files.

## Supplementary Materials

The following supporting information can be downloaded at: https://www.mdpi.com/article/10.3390/pharmaceutics15082140/s1. Figure S1: Cross-validated R2Y and Q2 coefficients and permutation test; Table S1: List of identified metabolites extracted from MS-DIAL; Table S2: Classification of metabolites formed from the ClassyFire system. Table S3: Pathway enrichment analysis of altered metabolites.
