# Supplementary material for: The Impact of Pigment-Epithelium-Derived Factor on MCF-7 Cell Metabolism in the Context of Glycaemic Condition
Source: Pharmaceutics. 2023 Aug 14;15(8):2140. doi: 10.3390/pharmaceutics15082140 (PMC10459545; doi:10.3390/pharmaceutics15082140)
Supplement: Supplementary file 1 [file pharmaceutics-15-02140-s001.zip › pharmaceutics-2511821-supplementary.pdf]

The Impact of Pigment-Epithelium-Derived Factor on MCF-7 Cell Metabolism in the Context of Glycaemic Condition

Raziyeh Abooshahab [1,2], Kourosh Hooshmand [3], Hani-Al Salami [1,4] and Crispin R. Dass [1,2,*]

[1]Curtin Medical School, Curtin University, Bentley, WA 6102, Australia

[2]Curtin Health Innovation Research Institute, Curtin Medical School, Curtin University, Bentley, WA 6102, Australia

[3]System Medicine, Steno Diabetes Center Copenhagen, 2730 Copenhagen, Denmark

[4]Biotechnology and Drug Development Research Laboratory, Curtin Health Innovation Research Institute, Bentley, WA 6102, Australia

** Corresponding author*

Crispin R. Dass (PhD), Professor,

Curtin Medical School, Curtin University, Bentley 6102, Australia.

Office: Curtin Health Innovation Research Institute, Bldg 305, Room 124, Curtin University, Bentley campus.

Phone: +61 8 9266 1489

E-mail: crispin.dass@curtin.edu.au

Supplemental Figure S1 Cross-validated R2Y and Q2 coefficients and permutation test.

Supplemental Table S1 List of identified metabolites extracted from MS-DIAL

Supplemental Table S2 Classification of metabolites formed from the ClassyFire system

Supplemental Table S3 Pathway Enrichment analysis of altered metabolites

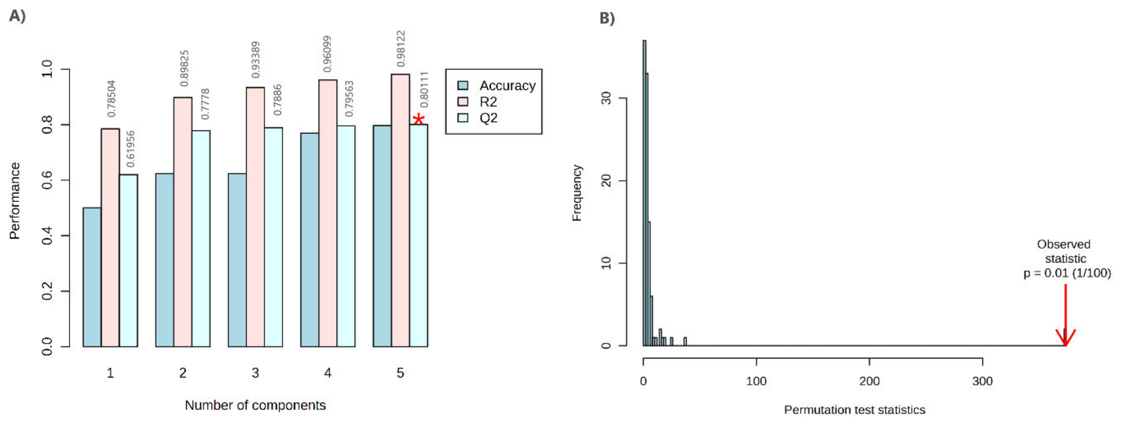

Figure S1: A) Cross-validated R2Y and Q2 coefficients. The red asterisk implies the best classifier. **B)** Based on the histogram displaying the permutation test results with a permutation number of n=100, it is clear that the model has been determined to be significant.

Table S1 List of identified metabolites extracted from MS-DIAL

Metabolite name	Average Rt(min)	Average RI	Quant mass
**1-Methylhistamine**	10.085	1738.14	174.1294
**1-Monopalmitin**	14	2584.1	371.4
**3-Methylglutaconic acid**	8.773	1512.32	191.12
**Aminomalonic acid**	8.571	1481.78	147.0867
**Arachidonic acid**	13.157	2385.64	79.0125
**beta-Alanine**	8.288	1441.1	174.1
**Cholesterol**	17.216	3263.57	129.1
**Citric acid**	10.62	1843.05	73.1
**Cysteine**	9.109	1568.69	69
**Dihydrouracil**	6.221	1150.24	73.08333
**3-Hydroxybutyric acid**	6.272	1156.72	191.1143
**Homocysteine**	9.746	1676.32	234.1231
**D-Threitol**	8.811	1518.49	217.1429
**Ethanolamine**	7.188	1286.33	174.1167
**Fumaric acid**	7.574	1340.79	117.0667
**Glycerol**	7.227	1290.2	73.1
**Glycine**	7.484	1328.16	174.1833
**Hypotaurine**	9.403	1618.09	156.05
**Arabitol**	10.145	1749.89	69.07895
**Malate**	8.657	1494.04	147.0846
**Proline**	7.419	1318.96	142.1046
**Threonine**	8.02	1403.65	73.1
**Lactic acid**	5.575	1043.76	73.1
**Lysine**	11.142	1945.45	174.15
**Oxoproline**	8.92	1537.04	156.112
**Alanine**	5.918	1106.82	116.1
**Aspartic acid**	8.254	1437.24	75
**Glutamic acid**	9.46	1627.64	246.2053
**Isoleucine**	7.21	1289.43	158.1913
**Methionine**	8.886	1531.21	176.125
**Ornithine**	10.614	1842	142.1

**Phenylalanine**	9.563	1645.04	73.09583
**Serine**	7.827	1376.54	73.1
**Tyrosine**	11.243	1965.28	218.175
**Valine**	6.472	1185.12	73.1
**Malonic acid**	6.426	1178.74	147.1
**Maltose**	14.656	2742.47	73.1
**Melibiose**	14.736	2761.82	73.1
**Methylmalonic acid**	6.609	1204.44	147.0955
**myo-Inositol**	12.073	2133.36	202.1
**Oleic acid**	12.473	2226.03	73.1
**O-Phosphoethanolamine**	10.447	1809.17	73.09
**Palmitic acid**	11.669	2048.86	73.1
**Palmitoleic Acid**	11.586	2032.5	117.0333
**Pimelic acid**	9.351	1609.43	149.0444
**Stearic acid**	12.572	2251.34	341.4
**Succinic acid**	7.485	1326.34	147.1
**Uracil**	7.812	1374.52	241.1

RT retention time, RI retention indices

Table S2 Classification of metabolites formed from the ClassyFire system

InChIKey	Kingdom	Superclass	Class	Subclass	Parent Level 1	Parent Level 2	Parent Level 3	Parent Level 4
FHQDWPCFSJMNCT-UHFFFAOYSA-N	Organic compounds	Organic nitrogen compounds	Organonitrogen compounds	Amines	Primary amines	2-arylethylamines		
LUHPQMTVPLRAQA-UHFFFAOYSA-N	Organic compounds	Lipids and lipid-like molecules	Fatty Acyls	Fatty acid esters	Fatty acid esters			
LVVVFIKBESNWRW-CSKARUKUSA-N	Organic compounds	Organometallic compounds	Organometalloid compounds	Organosilicon compounds	Trimethylsilyl esters			
HFJBTSWFKQDXSU-UHFFFAOYSA-N	Organic compounds	Organic acids and derivatives	Carboxylic acids and derivatives	Amino acids, peptides, and analogues	Amino acids and derivatives	Alpha amino acids and derivatives		
YZXBAPSDXZZRGB-DOFZRALJSA-N	Organic compounds	Lipids and lipid-like molecules	Fatty Acyls	Fatty acids and conjugates	Long-chain fatty acids			
UCMIRNVEIXFBKS-UHFFFAOYSA-N	Organic compounds	Organic acids and derivatives	Carboxylic acids and derivatives	Amino acids, peptides, and analogues	Amino acids and derivatives	Beta amino acids and derivatives		
SGNBVLSWZMBQTH-PODYLUTMSA-N	Organic compounds	Lipids and lipid-like molecules	Steroids and steroid derivatives	Ergostane steroids	Ergosterols and derivatives			
KRKNYBCHXYNGOX-UHFFFAOYSA-N	Organic compounds	Organic acids and derivatives	Carboxylic acids and derivatives	Tricarboxylic acids and derivatives	Tricarboxylic acids and derivatives			
XUJNEKJLAYXESH-UHFFFAOYNA-N	Organic compounds	Organic acids and derivatives	Carboxylic acids and derivatives	Amino acids, peptides, and analogues	Amino acids and derivatives	Alpha amino acids and derivatives	Cysteine and derivatives	

OIVLITBTBDPEFK-UHFFFAOYSA-N	Organic compounds	Organoheterocyclic compounds	Diazines	Pyrimidines and pyrimidine derivatives	Pyrimidones			
WHBMMWSBFZVSSR-UHFFFAOYSA-N	Organic compounds	Organic acids and derivatives	Hydroxy acids and derivatives	Beta hydroxy acids and derivatives	Beta hydroxy acids and derivatives			
FFFHZYDWPBMWHY-UHFFFAOYSA-N	Organic compounds	Organic acids and derivatives	Carboxylic acids and derivatives	Amino acids, peptides, and analogues	Amino acids and derivatives	Alpha amino acids and derivatives	Alpha amino acids	
UNXHWFMMPAWVPI-QWWZWVQMSA-N	Organic compounds	Organic oxygen compounds	Organooxygen compounds	Carbohydrates and carbohydrate conjugates	Sugar alcohols			
MZTOEZRSUGVLOG-UHFFFAOYSA-N	Organic compounds	Organometallic compounds	Organometalloid compounds	Organosilicon compounds	Organoheterosilanes	Trialkylheterosilanes		
VZCYOOQTPOCHFL-OWOJBTEDSA-N	Organic compounds	Organic acids and derivatives	Carboxylic acids and derivatives	Dicarboxylic acids and derivatives	Dicarboxylic acids and derivatives			
JQUGYGVCECHKBA-UHFFFAOYSA-N	Organic compounds	Organometallic compounds	Organometalloid compounds	Organosilicon compounds	Organoheterosilanes	Trialkylheterosilanes		
DHMQDGOQFOQNFH-UHFFFAOYSA-N	Organic compounds	Organic acids and derivatives	Carboxylic acids and derivatives	Amino acids, peptides, and analogues	Amino acids and derivatives	Alpha amino acids and derivatives	Alpha amino acids	
VVIUBCNYACGLLV-UHFFFAOYSA-N	Organic compounds	Organic acids and derivatives	Sulfinic acids and derivatives	Sulfinic acids	Sulfinic acids			
SUZLPERYXSOGNY-UHFFFAOYSA-N	Organic compounds	Organic oxygen compounds	Organooxygen compounds	Carbohydrates and carbohydrate conjugates	Monosaccharides			

BJEPYKJPYRNKOW-REOHCLBHSA-N	Organic compounds	Organic acids and derivatives	Hydroxy acids and derivatives	Beta hydroxy acids and derivatives	Beta hydroxy acids and derivatives			
ONIBWKKTOPOVIA-BYPYZUCNSA-N	Organic compounds	Organic acids and derivatives	Carboxylic acids and derivatives	Amino acids, peptides, and analogues	Amino acids and derivatives	Alpha amino acids and derivatives	Proline and derivatives	
AYFVYJQAPQTCCC-GBXIJSLDSA-N	Organic compounds	Organic acids and derivatives	Carboxylic acids and derivatives	Amino acids, peptides, and analogues	Amino acids and derivatives	Alpha amino acids and derivatives	Alpha amino acids	L-alpha-amino acids
JVTAAEKCZFNVCJ-REOHCLBHSA-N	Organic compounds	Organic acids and derivatives	Hydroxy acids and derivatives	Alpha hydroxy acids and derivatives	Alpha hydroxy acids and derivatives			
KDXKERNSBIXSRK-YFKPBYRVSA-N	Organic compounds	Organic acids and derivatives	Carboxylic acids and derivatives	Amino acids, peptides, and analogues	Amino acids and derivatives	Alpha amino acids and derivatives	Alpha amino acids	L-alpha-amino acids
QACGFKAUSXGLCU-UHFFFAOYSA-N	Organic compounds	Organic acids and derivatives	Carboxylic acids and derivatives	Amino acids, peptides, and analogues	Amino acids and derivatives	Alpha amino acids and derivatives	Proline and derivatives	
QNAYBMKLOCPYGJ-REOHCLBHSA-N	Organic compounds	Organic acids and derivatives	Carboxylic acids and derivatives	Amino acids, peptides, and analogues	Amino acids and derivatives	Alpha amino acids and derivatives	Alanine and derivatives	
UTGZLLJGNUQPMX-UHFFFAOYSA-N	Organic compounds	Organic acids and derivatives	Carboxylic acids and derivatives	Amino acids, peptides, and analogues	Amino acids and derivatives	Alpha amino acids and derivatives	Aspartic acid and derivatives	
WHUUTDBJXJRKMK-VKHMYHEASA-N	Organic compounds	Organic acids and derivatives	Carboxylic acids and derivatives	Amino acids, peptides, and analogues	Amino acids and derivatives	Alpha amino acids and derivatives	Glutamic acid and derivatives	

AGPKZVBTJJNPAG-WHFBIAKZSA-N	Organic compounds	Organic acids and derivatives	Carboxylic acids and derivatives	Amino acids, peptides, and analogues	Amino acids and derivatives	Alpha amino acids and derivatives	Isoleucine and derivatives	
FFEARJCKVFRZRR-BYPYZUCNSA-N	Organic compounds	Organic acids and derivatives	Carboxylic acids and derivatives	Amino acids, peptides, and analogues	Amino acids and derivatives	Alpha amino acids and derivatives	Methionine and derivatives	
AHLPHDHHMVZTML-BYPYZUCNSA-N	Organic compounds	Organic acids and derivatives	Carboxylic acids and derivatives	Amino acids, peptides, and analogues	Amino acids and derivatives	Alpha amino acids and derivatives	Alpha amino acids	L-alpha-amino acids
COLNVLDHVKWLRT-QMMMGPOBSA-N	Organic compounds	Organic acids and derivatives	Carboxylic acids and derivatives	Amino acids, peptides, and analogues	Amino acids and derivatives	Alpha amino acids and derivatives	Phenylalanine and derivatives	
MTCFGRXMJLQNBG-REOHCLBHSA-N	Organic compounds	Organic acids and derivatives	Carboxylic acids and derivatives	Amino acids, peptides, and analogues	Amino acids and derivatives	Alpha amino acids and derivatives	Serine and derivatives	
WMWBCQXPKSQMOK-KRWDZBQOSA-N	Organic compounds	Organic acids and derivatives	Carboxylic acids and derivatives	Amino acids, peptides, and analogues	Amino acids and derivatives	Alpha amino acids and derivatives	Phenylalanine and derivatives	
KZSNJWFQEVHDMF-BYPYZUCNSA-N	Organic compounds	Organic acids and derivatives	Carboxylic acids and derivatives	Amino acids, peptides, and analogues	Amino acids and derivatives	Alpha amino acids and derivatives	Valine and derivatives	
OFOBLEOULBTSOW-UHFFFAOYSA-N	Organic compounds	Organic acids and derivatives	Carboxylic acids and derivatives	Dicarboxylic acids and derivatives	Dicarboxylic acids and derivatives			
GUBGYTABKSRVRQ-PICCSMPSSA-N	Organic compounds	Organic oxygen compounds	Organooxygen compounds	Carbohydrates and carbohydrate conjugates	Glycosyl compounds	O-glycosyl compounds		

DLRVVLDZNNYCBX-CQHUIXDMSA-N	Organic compounds	Organic oxygen compounds	Organooxygen compounds	Carbohydrates and carbohydrate conjugates	Glycosyl compounds	O-glycosyl compounds		
ZIYVHBGGAOATLY-UHFFFAOYSA-N	Organic compounds	Organic acids and derivatives	Carboxylic acids and derivatives	Dicarboxylic acids and derivatives	Dicarboxylic acids and derivatives			
CDAISMWEOUEBRE-GPIVLXJGSA-N	Organic compounds	Organic oxygen compounds	Organooxygen compounds	Alcohols and polyols	Secondary alcohols	Cyclohexanols		
ZQPPMHVWECSIRJ-KTKRTIGZSA-N	Organic compounds	Lipids and lipid-like molecules	Fatty Acyls	Fatty acids and conjugates	Long-chain fatty acids			
SUHOOTKUPISOBE-UHFFFAOYSA-N	Organic compounds	Organic acids and derivatives	Organic phosphoric acids and derivatives	Phosphate esters	Phosphoethanolamines			
IPCSVZSSVZVIGE-UHFFFAOYSA-N	Organic compounds	Lipids and lipid-like molecules	Fatty Acyls	Fatty acids and conjugates	Long-chain fatty acids			
UNUADFKHVGBUHA-KHPPLWFESA-N	Organic compounds	Organometallic compounds	Organometalloid compounds	Organosilicon compounds	Trimethylsilyl esters			
VOLKRTUGJXKFMI-UHFFFAOYSA-N	Organic compounds	Organometallic compounds	Organometalloid compounds	Organosilicon compounds	Trimethylsilyl esters			
QIQXTHQIDYTFRH-UHFFFAOYSA-N	Organic compounds	Lipids and lipid-like molecules	Fatty Acyls	Fatty acids and conjugates	Long-chain fatty acids			
KDYFGRWQOYBRFD-UHFFFAOYSA-N	Organic compounds	Organic acids and derivatives	Carboxylic acids and derivatives	Dicarboxylic acids and derivatives	Dicarboxylic acids and derivatives			
FSBNTQRWSOTNEW-UHFFFAOYSA-N	Organic compounds	Organoheterocyclic compounds	Diazines	Pyrimidines and pyrimidine derivatives	Pyrimidines and pyrimidine derivatives			

Table S3: Pathway enrichment analysis of altered metabolites

Metabolic Pathways	[a]Total	[b]Hits	[c]Raw p	[d]FDR
Arginine and Proline Metabolism	53	4	0.00233	0.128
Glycine and Serine Metabolism	59	4	0.00348	0.128
Citric Acid Cycle	32	3	0.00504	0.128
Beta-Alanine Metabolism	34	3	0.006	0.128
Aspartate Metabolism	35	3	0.00652	0.128
Methionine Metabolism	43	3	0.0117	0.19
Glutamate Metabolism	49	3	0.0167	0.234
Mitochondrial Electron Transport Chain	19	2	0.0193	0.235
Betaine Metabolism	21	2	0.0234	0.235
Carnitine Synthesis	22	2	0.0255	0.235
Warburg Effect	58	3	0.0263	0.235
Urea Cycle	29	2	0.0429	0.35
Purine Metabolism	74	3	0.0497	0.36
Ammonia Recycling	32	2	0.0514	0.36
Propanoate Metabolism	42	2	0.0836	0.546
Homocysteine Degradation	9	1	0.101	0.619
Malate-Aspartate Shuttle	10	1	0.112	0.644
Ketone Body Metabolism	13	1	0.143	0.714
Pyrimidine Metabolism	59	2	0.149	0.714
Valine, Leucine and Isoleucine Degradation	60	2	0.153	0.714
Vitamin K Metabolism	14	1	0.153	0.714
Bile Acid Biosynthesis	65	2	0.174	0.741
Alanine Metabolism	17	1	0.183	0.741
Spermidine and Spermine Biosynthesis	18	1	0.193	0.741
Butyrate Metabolism	19	1	0.202	0.741
Tyrosine Metabolism	72	2	0.204	0.741
Catecholamine Biosynthesis	20	1	0.212	0.741
Threonine and 2-Oxobutanoate Degradation	20	1	0.212	0.741
Glutathione Metabolism	21	1	0.221	0.748
Transfer of Acetyl Groups into Mitochondria	22	1	0.231	0.753
Oxidation of Branched Chain Fatty Acids	26	1	0.267	0.817
Phytanic Acid Peroxisomal Oxidation	26	1	0.267	0.817
Phenylalanine and Tyrosine Metabolism	28	1	0.284	0.844
Porphyrin Metabolism	40	1	0.382	1
Histidine Metabolism	43	1	0.404	1
Steroidogenesis	43	1	0.404	1
Steroid Biosynthesis	48	1	0.44	1
Pyruvate Metabolism	48	1	0.44	1

[a] Total Cmpd: total number of compounds in the pathway, [b] Hit: actually matched number from the data

[c] Raw p: p-value calculated from the enrichment analysis, [d] FDR: p-value adjusted using False Discovery Rate
